# Study of the American Medicinal Flora

**Published:** 1897-09

**Authors:** 


### STUDY OF THE AMERICAN MEDICINAL FLORA.

NEW YORK COLLEGE OF PHARMACY, N. Y., June 25, 1897.

*Atlanta Medical and Surgical Journal:*

The Sub-Commission of the Pan-American Medical Congress appointed to study the medicinal plants of the United States has entered into an association with the Smithsonian Institution for that purpose. The attention of your readers is called to the respective circulars issued by these organizations.

SMITHSONIAN INSTITUTION, WASHINGTON, D. C.
May 28, 1897.

DEAR SIR:—The Smithsonian Institution has undertaken to bring together all possible material bearing on the medicinal uses of plants in the United States. Arrangements have been made with a body representing the Pan-American Medical Congress, the Sub-Commission on Medicinal Flora of the United States, to elaborate a report on this subject, and the material when received will be turned over to them for investigation.

The accompanying detailed instructions relative to specimens and notes have been prepared by the Sub-Commission.

All packages and correspondence should be addressed to the "Smithsonian Institution, Washington, D. C.," and marked on the outside "Medicinal Plants, for the U. S. National Museum."

Franks which will carry specimens, when of suitable size, together with descriptions and notes, free of postage through the mails, will be forwarded upon application. Should an object be too large for transmission by mail, the sender is requested, before shipping it, to notify the Institution, in order that a proper authorization for its shipment may be made out.

Respectfully,

S. P. LANGLEY, Secretary.

INSTRUCTIONS RELATIVE TO MEDICINAL PLANTS.

The Pan-American Medical Congress, at its meeting held in the City of Mexico in November, 1896, took steps to institute a systematic study of the American medicinal flora, through the medium of a General Commission and of special Sub-Commissions, the latter to be organized in the several countries. The Sub-Commission for the United States has been formed and consists of Dr. Valery Harvard, U. S. A., Chairman; Mr. Frederick V. Colville, Botanist of the United States Department of Agriculture; Dr. C. F. Millspaugh, Curator of the Botanical Department of the Field Columbian Museum, Chicago; Dr. Charles Mohr, State Botanist of Alabama; Dr. W. P. Wilson, Director of the Philadelphia Commercial Museums; and Prof. H. H. Rusby, of the New York College of Pharmacy. This Sub-Commission solicits information concerning the medicinal plants of the United States from every one in a position to accord it. The principal points of study are as follows:

1. Local names. 2. Local uses, together with historical facts. 3. Geographical distribution and degree of abundance in the wild state. 4. Is the plant collected for market, and if so (*a*) at what season of the year? (*b*) To how great an extent? (*c*) How prepared for market? (*d*) what is the effect of such collection upon the wild supply? (*e*) What price does it bring? (*f*) Is the industry profitable? 5. Is the plant, or has it ever been, cultivated, and if so give all information on the subject, particularly as to whether such supplies are of superior quality, and whether the industry has proved profitable. 6. If not cultivated, present facts concerning the life-history of the plant which might aid in determining methods of cultivation. 7. Is the drug subjected to substitution or adulteration, and if so, give information as to the plants used for this purpose.

While it is not expected that many persons will be able to contribute information on all these points concerning any plant, it is hoped that a large number of persons will be willing to communicate such partial knowledge as they possess.

In order to assist in the study of the habits, properties and uses of medicinal plants, the Sub-Commission undertakes to furnish the name of any plant-specimen received, together with any desired information available.

Owing to the diversity in the common names of many plants it will be necessary for reports, when not furnished by botanists or others qualified to state the botanical names with certainty, to accompany the same with some specimen of the plant sufficient for its identification. While the Sub-Commission will endeavor to determine the plant from any portion of it which may be sent, it should be appreciated that the labor of identification is very greatly decreased, and its usefulness increased, by the possession of complete material, that is, leaf, flower and fruit, and in the case of small plants, the underground portion also. It is best to dry such specimens thoroughly, in a flat condition under pressure, before mailing. While any convenient means for accomplishing this result may be employed, the following procedure is recommended. Select a flowering or fruiting branch, as the case may be, which, when pressed, shall not exceed sixteen inches in length by ten inches in width. If the plant be a herb two or three feet high, it may be doubled to bring it within these measurements. If it possesses root leaves, some of these should be included. Lay the specimen flat in a fold of newspaper and place this in a pile of newspapers, carpet felting, or some other form of paper which readily absorbs moisture, and place the pile in a dry place, under a pressure of about twenty to thirty pounds, sufficient to keep the leaves from wrinkling as they dry. If a number of specimens are pressed at the same time, each is to be separated from the others by three or four folded newspapers or an equivalent in other kinds of paper. In twelve to twenty-four hours these papers will be found saturated with the absorbed moisture, and the fold containing the specimens should be transferred to dry ones. This change should be repeated for from two to five days, according to the state of the weather, the place where the drying is done, the fleshiness of the specimens, etc. The best way to secure the required pressure is by means of a pair of strong straps, though weights will do. The best place for drying is beside a hot kitchen range. When dry the specimens should be mailed between cardboards, or some other light but stiff materials which will not bend in transit.

It is a most important matter that the name and address of the sender should be attached to the package and that the specimens, if more than one, should be numbered, the sender retaining also specimens bearing the same number, to facilitate any correspondence which may follow. The sub-commission requests that, so far as practicable, all plants sent be represented by at least four specimens. (Signed) H. H. RUSBY, M.D.,

Chairman of the Gen. Com., New York College of Phar.

VALERY HARVARD, M.D.,

Chairman of the Sub-Com., Fort Slocum, Davids Island, N. Y.

---

I always take in hand seriously the undergraduate who expresses his intention of paying particular attention to a certain branch with a view to making it a specialty when he shall have graduated. It is this kind of specialist who, as much as the advertising quack, has brought the very word into disrepute. I tell my young friend that it will be time enough when he has thoroughly grounded himself in the general principles and practice of his profession and has had ten or fifteen years of experience, to think of devoting his time in great part to some one line more than others. I would not discourage any young practitioner from endeavoring to increase his knowledge and perfect his skill in certain particular lines or line, according as he may have talent, taste or opportunity to study in that direction, for the field has become too wide for one to become expert in everything, and with quaint old Norris, "I think a little plot of ground thick sown better than a great field, which, for the most part, lies fallow." If by and by a man becomes wise or skilful beyond his fellows in a certain line of work, and they keep him so busy therein that he has no time for anything else, I can see no objection to his doing that work, whether he be called a specialist or whether he is called an expert in that line. And if all specialists were made in this way there would be no cause of complaint from anybody.—*Dr. J. W. Kelly, N. O. Med. and Surg. Jour.*

